# The prognostic value of admission D-dimer level in patients with cardiogenic shock after acute myocardial infarction

**DOI:** 10.3389/fcvm.2022.1083881

**Published:** 2023-01-09

**Authors:** Yi Jiang, Yuansong Zhu, Zhenxian Xiang, Bryan Richard Sasmita, Yaxin Wang, Gong Ming, Siyu Chen, Suxin Luo, Bi Huang

**Affiliations:** Department of Cardiology, The First Affiliated Hospital of Chongqing Medical University, Chongqing, China

**Keywords:** cardiogenic shock, acute myocardial infarction, D-dimer, risk score, short-term outcome

## Abstract

**Background:**

Shock is associated with the activation of the coagulation and fibrinolytic system, and D-dimer is the degradation product of cross-linked fibrin. However, the prognostic value of D-dimer in patients with cardiogenic shock (CS) after acute myocardial infarction (AMI) remains unclear.

**Methods:**

We retrospectively analyzed the data of consecutive patients with CS complicating AMI. The primary endpoint was 30-day mortality and the secondary endpoint was the major adverse cardiovascular events (MACEs) including 30-day all-cause mortality, ventricular tachycardia/ventricular fibrillation, atrioventricular block, gastrointestinal hemorrhage, and non-fatal stroke. Restricted cubic spline (RCS) analyses were performed to assess the association between admission D-dimer and outcomes. A multivariable Cox regression model was performed to identify independent risk factors. The risk predictive potency with D-dimer added to the traditional risk scores was evaluated by C-statistics and the net reclassification index.

**Results:**

Among 218 patients with CS complicating AMI, those who died during the 30-day follow-up presented with worse baseline characteristics and laboratory test results, including a higher level of D-dimer. According to the X-tile program result, the continuous plasma D-dimer level was divided into three gradients. The 30-day all-cause mortality in patients with low, medium, and high levels of D-dimer were 22.4, 53.3, and 86.2%, respectively (*p* < 0.001 for all). The 30-day incidence of MACEs was 46.3, 77.0, and 89.7%, respectively (*p* < 0.001). In the multivariable Cox regression model, the trilogy of D-dimer level was an independent risk predictor for 30-day mortality (median D-dimer cohort: HR 1.768, 95% CI 0.982–3.183, *p* = 0.057; high D-dimer cohort: HR 2.602, 95% CI 1.310–5.168, *p* = 0.006), a similar result was observed in secondary endpoint events (median D-dimer cohort: HR 2.012, 95% CI 1.329–3.044, *p* = 0.001; high D-dimer cohort: HR 2.543, 95% CI 1.452–4.453, *p* = 0.001). The RCS analyses suggested non-linear associations of D-dimer with 30-day mortality. The enrollment of D-dimer improved risk discrimination for all-cause death when combined with the traditional CardShock score (C-index: 0.741 vs. 0.756, *p*_difference_ = 0.004) and the IABP-SHOCK II score (C-index: 0.732 vs. 0.754, *p*_difference_ = 0.006), and the GRACE score (C-index: 0.679 vs. 0.715, *p*_difference_ < 0.001). Similar results were acquired after logarithmic transformed D-dimer was included in the risk score. The improvements in reclassification which were calculated as additional net reclassification index were 7.5, 8.6, and 12.8%, respectively.

**Conclusion:**

Admission D-dimer level was independently associated with the short-term outcome in patients with CS complicating AMI and addition of D-dimer brought incremental risk prediction value to traditional risk prediction scores.

## Introduction

Cardiogenic shock (CS) represents a critical hypoperfusion status resulting from cardiac output failing to meet the metabolic demands of multiple organs and the initial insult can be primarily attributed to cardiac dysfunction. Among the broad spectrum of etiologies, ventricular failure subsequent to acute myocardial infarction (AMI) remains the most frequent cause of CS (1).

Previous studies have shown that shock, regardless of the etiologies, is associated with the activation of coagulation and fibrinolysis (2). D-dimer is the degradation product of cross-linked fibrin, reflecting both thrombin production and the activation of fibrinolysis. Traditionally, atherothrombosis is regarded as the activation of platelets, while venous thromboses with coagulation dysfunction. However, an increasing body of evidence suggested that these two morbidities shared similar pathogenic pathways (3–5). Consequently, increased fibrin turnover is found during atherothrombosis (6).

Previous studies have shown that D-dimer provided risk stratification information for patients with AMI and the elevation of the D-dimer was associated with increased mortality in patients with AMI (7–10). However, the prognostic value of D-dimer has not been well-understood in patients with CS complicating AMI. In the present study, we aimed to evaluate the association of D-dimer with short-term prognosis in patients with CS complicating AMI and whether D-dimer could improve the risk prediction power based on the established risk score system.

## Materials and methods

### Study design

This retrospective observational study was performed in a single tertiary care institute (The first affiliated hospital of Chongqing medical university, Chongqing, China). Patients diagnosed with CS complicating AMI from January 2013 to September 2020 were enrolled and data included baseline characteristics, laboratory findings. The result of auxiliary examination were extracted from the electrical medical record system of our institution (the Classification of Diseases, 10th Revision, Clinical Modification were used to identify patients). The patients were followed by phone calls or clinical visits. To ensure data accuracy, the diagnosis of all events was reviewed by experienced cardiac physicians. The research protocol was approved by the ethics committee of the first affiliated hospital of Chongqing Medical University.

### Definition

Diagnoses and classifications of AMI and CS were made in line with universal definitions up to date. The diagnosis of AMI was made according to the fourth universal definition of myocardial infarction (11). After reviewing and careful evaluation, the diagnosis of CS was made as sustained systolic blood pressure (SBP) < 90 mmHg and cardiac index < 2.2 L/min/m^2^ with adequate volume load, combined with clinical or laboratory signs of hypoperfusion, or the requirement for inotropic or vasopressor agents or mechanical circulation support to maintain blood pressure and cardiac index. Clinical signs of hypoperfusion include cold extremities, oliguria, mental confusion, dizziness, and narrow pulse pressure, and laboratory findings include metabolic acidosis, elevated serum lactate, and serum creatinine (12–15).

Definitions of events are as follows. All-cause mortality was defined as death from any cause. Arrhythmia was captured by means of electrocardiogram (ECG), Holter document, or electrocardiographic monitoring that was recorded in the medical records. In terms of ventricular tachycardia (VT), only sustained VT was included in our study, which was defined as a ventricular rhythm faster than 100 bpm lasting at least 30 s or requiring termination due to hemodynamic instability. Ventricular fibrillation (VF) was defined as loss of consciousness in patients without identifiable repetitive waveforms or intervals on ECG. Atrioventricular block (AVB) was defined as a delay or interruption in the transmission of an impulse from the atria to the ventricles. Both persistent and paroxysmal AVB were included in the present study. Non-fatal stroke was defined as focal neurologic signs thought to be of vascular origin that persisted for more than 24 hours, confirmed by computed tomographic scans or magnetic resonance imaging. Only symptomatic events were defined as events, and silent stroke was treated as an incidental finding. Gastrointestinal hemorrhage (GIH) was characterized as hematemesis, melena, or both with a hemoglobin decrease of at least 2 g/dL or leading to a transfusion of ≥2 units of blood.

### Coronary angiography and percutaneous coronary intervention (PCI) procedure

After taking a loading dose of dual antiplatelet drugs (aspirin 100 mg and ticagrelor 180 mg/clopidogrel 300 mg), patients were immediately transferred to the catheterization laboratory for emergency coronary angiography. According to the angiography results, the revascularization strategy was individualized by the interventionists. Blood flow of the infarct-related artery was assessed according to Thrombolysis in Myocardial Infarction (TIMI) grading system. The usage of relevant devices such as intra-aortic balloon pump (IABP) was at the discretion of the experienced interventionists. After the procedure, all patients were transferred to the coronary care unit for close monitoring.

### Laboratory and auxiliary tests

Blood samples for cardiac enzymes and arterial blood gas were collected as soon as patients were admitted to an emergency department and were analyzed at a central laboratory. D-dimer was measured in venous blood at hospital admission, using a kit device (Alere, Triage^®^ Meter) with a transfer pipette for bedside measurement of D-dimer.

### The clinical risk scores

The Intra-aortic Balloon Pump in Cardiogenic Shock (IABP-SHOCK II) score and the CardShock score have been described in previous reports (16, 17) and performed well in the validation analysis. The AUC of the CardShock score was 0.85 (95% CI: 0.79–0.90) and the AUC of the IABP-SHOCK II score was 0.79 (95% CI: 0.70–0.88), respectively. The GRACE score has excellent discrimination ability as reflected by the C statistic of 0.84 (18).

Clinical data required for calculating the risk scores were retrieved from medical records, including age, history of stroke, glucose, creatinine, arterial lactate, and TIMI flow grade after PCI.

### Endpoints

Since a previous study revealed that most of the adverse events occurred in the early period after AMI (1), we set 30-day all-cause mortality as the primary endpoint. The secondary endpoint was the major adverse cardiovascular events (MACEs) including 30-day all-cause mortality, VT/VF, AVB, GIH, or non-fatal stroke.

### Statistical analysis

Categorical variables were presented in numbers and percentages. Continuous variables which followed the normal distribution were presented in mean value and standard deviations, as median value, and interquartile range (25th and 75th) methods were employed for those who were not. Multiple imputations were performed for the missing values of lab test results.

Two independent sample *t*-test was used for comparisons of continuous variables, and the *Mann-Whitney U* test was used for those with a non-positive distribution. χ^2^ test or *Fisher* test was employed for categorical variables comparison. Survival analysis and cumulative incidence of endpoint events were assessed by *Kaplan-Meier* plot and *log-rank* test.

To visually assess the relationship between D-dimer and endpoints events, we used restricted cubic spline (RCS) with four knots at the 5th, 35th, 65th, and 95th centiles to flexibly model the relationship between D-dimer with all-cause mortality. D-dimer was transferred into logarithmic value to alleviate non-linearity. The optimal cut-off points were defined using the X-tile program (Rimm Lab, Yale School of Medicine).

The univariable Cox regression model was used to explore the relationship between D-dimer level and clinical outcomes. Based on the result of the univariable analysis, we took clinical relevance and model stability into consideration to decide which variables were selected for the multivariable Cox regression model.

The ability of risk discrimination was quantified by C-index, which was calculated before and after D-dimer was added to the risk score. The calibration curve was employed to visualize the agreement between model predictions and observation. Improvement in risk prediction was quantified by the reclassification index (NRI). Increases in predicted risk in cases and decreases in non-cases with a variation of more than 5% were regarded as improvements.

A two-tailed *p*-value of <0.05 was considered statistically significant in this study. All of the analyses were performed with the statistical software R V.3.6.1 (R Foundation for Statistical Computing, Vienna, Austria), and SPSS version 25.0 (IBM, USA).

## Results

### Baseline characteristics and outcomes

From January 2013 to September 2020, 245 patients were diagnosed with CS-complicating AMI in our institution, among which 27 patients were excluded due to incomplete data or did not undergo coronary angiography. Finally, 218 (89.0%) patients with complete data were analyzed in the present study.

The baseline characteristics were displayed in Table 1. Compared with survivors, the non-survivors tended to be older and female, but less likely to be a drinker or smoker. The non-survivors were more likely to have a history of PCI and hypertension. On admission, the non-survivors presented with lower systolic blood pressure and a higher level of lactate, B-type natriuretic peptide, white blood cell count, creatinine, procalcitonin, and D-dimer (all *p* < 0.05). The latter group also had relatively lower albumin, prothrombin activity, and left ventricular ejection fraction (all *p* < 0.05). There was no significant difference in terms of myocardial infarction location between survivors and non-survivors. The risk scores (CardShock, IABP-SHOCK II, GRACE) in non-survivors were significantly higher than in survivors. Stent implantation was performed more frequently in the survivors while the non-survivors were more likely to receive ventilation support (all *p* < 0.001). As for medication use, except for dopamine and P2Y12 inhibitors, other medications were well-balanced between survival and non-survival patients.

**TABLE 1:** Comparison of baseline characteristics stratified by the primary outcome.

	Survivors	Non-survivors	*P*-value
	(*N* = 113)	(*N* = 105)	
Age (years)	66.5 ± 11.2	74.2 ± 10.2	<0.001
Men	80 (70.8%)	61 (58.1%)	0.05
Body mass index (Kg/m^2^)	23.6 ± 2.8	22.6 ± 3.1	0.016
Alcohol use	45 (39.8%)	27 (25.7%)	0.027
Smoker	73 (64.6%)	42 (40.0%)	<0.001
**Medical history**			
Hypertension	49 (43.4%)	60 (57.1%)	0.042
Diabetes	33 (29.2%)	40 (38.1%)	0.165
Coronary artery disease	18 (15.9%)	22 (21.0%)	0.338
Myocardial infarction	3 (2.7%)	9 (8.6%)	0.056
Percutaneous coronary intervention	5 (4.4%)	13 (12.4%)	0.033
Coronary artery bypass graft	0	3 (2.9%)	0.11
Stroke	5 (4.4%)	10 (9.5%)	0.137
Atrial fibrillation	6 (5.3%)	7 (6.7%)	0.672
Heart failure	4 (3.5%)	7 (6.7%)	0.292
**Vital signs**			
Systolic blood pressure (mmHg)	87 (79.0–99.0)	84 (78.0–98.5)	0.016
Diastolic blood pressure (mmHg)	57 (51.0–64.0)	55 (48.5–60.5)	0.17
Heart rate (bpm)	84.1 ± 27.8	91.4 ± 28.0	0.056
**Myocardial infarction location**			
Anterior	39 (34.5%)	41 (39.0%)	0.488
Inferior or posterior	53 (46.9%)	41 (39.0%)	0.242
Lateral	7 (6.2%)	11 (10.5%)	0.251
Right ventricle	18 (15.9%)	9 (8.6%)	0.099
**Laboratory and echocardiography finding**			
Lactate (mmol/L)	2.3 (1.6–3.6)	5.9 (2.95–8.6)	<0.001
Troponin I (ng/ml)	3.7 (0.7–9.8)	4.9 (1.3–16.0)	0.15
Brain natriuretic peptide (pg/ml)	216 (91.8–1,266.0)	2,130 (498.5–3,844.0)	<0.001
D-dimer (ng/ml)	780 (268–1,810)	1,760 (855–3,315)	<0.001
White blood cell (*109)	8.2 (6.6–10.8)	13.3 (10.1–18.1)	<0.001
Neutrophil (%)	73.4 ± 10.6	82.5 ± 11.7	<0.001
Creatinine (μmol/L)	94 (75–121)	144 (97–228)	<0.001
Procalcitonin (μg/L)	0.08 (0.05–1.42)	0.54 (0.14–3.58)	0.004
Albumin (g/L)	35.7 ± 5.6	32.1 ± 6.8	<0.001
Left ventricular ejection fraction (%)	51.6 ± 9.0	45.0 ± 9.5	<0.001
Prothrombin activity (%)	96.7 ± 25.8	72.8 ± 29.7	<0.001
**Risk scores**			
CardShock	4.1 ± 1.2	4.5 ± 1.4	0.010
IABP-SHOCK II	2.4 ± 1.6	3.7 ± 1.6	<0.001
GRACE	212.5 ± 25.7	230.7 ± 20.5	<0.001
**Treatment**			
Aspirin	106 (93.8%)	95 (90.5%)	0.36
P2Y12 inhibitor *	111 (98.2%)	92 (87.6%)	0.002
Dopamine	51 (45.1%)	65 (61.9%)	0.013
Nitrates	34 (30.1%)	29 (27.6%)	0.688
Digitalis	14 (12.4%)	14 (13.3%)	0.835
Stenting	95 (84.1%)	53 (50.5%)	<0.001
Intra-aortic balloon pump	20 (17.7%)	16 (15.2%)	0.625
Ventilation support	29 (25.7%)	69 (65.7%)	<0.001
Hemofiltration	0	3 (2.9%)	0.11
Anticoagulation	33 (29.2%)	24 (22.9%)	0.287

*Including clopidogrel and ticagrelor.

According to the cut-off values of D-dimer derived from the X-tile program, patients were divided into three groups, low D-dimer group (<720 ng/ml), median D-dimer group (720–3,600 ng/ml), and high D-dimer group (>3,600 ng/ml). The baseline characteristics and comparisons among the three groups were displayed in Supplementary Table 1. Generally, patients with high D-dimer levels tended to be older and presented with higher risk scores, lactate, B-type natriuretic peptide, and white blood cell count. Moreover, there was a lower rate of stent implantation in patients with high D-dimer levels.

### Associations between D-dimer and outcomes

**FIGURE 1:**
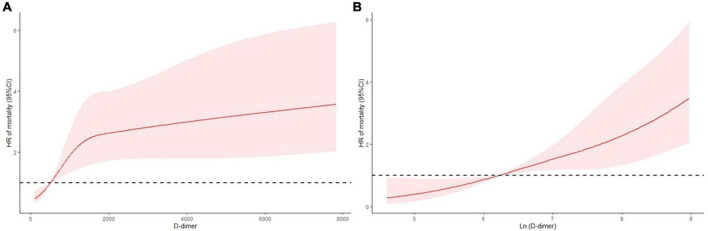
K-M curves for 30-day all-cause mortality and secondary endpoint events according to low (<720 ng/ml), median (720–3,600 ng/ml), and high (>3,600 ng/ml) levels of D-dimer. **(A)** All-cause mortality, **(B)** secondary endpoint events.

The primary and secondary endpoints are shown in Table 2. The 30-day all-cause mortality and MACEs incidence increased with D-dimer increase (all *p* < 0.001). The Kaplan-Meier curves are shown in Figure 1. As shown, compared with patients with low D-dimer, patients with median and high D-dimer had a significantly increased risk of 30-day mortality (Figure 1A). Also, as D-dimer increased, the rate of free from MACEs significantly decreased (Figure 1B).

**TABLE 2:** Occurrence of endpoint events stratified by D-dimer level.

	D-dimer <720 vs. >3,600 ng/ml			D-dimer <720 vs. 720–3,600 ng/ml			D-dimer 720–3,600 vs. >3,600 ng/ml		
	Low D-dimer	High D-dimer	*P*-value	Low D-dimer	Median D-dimer	*P*-value	Median D-dimer	High D-dimer	*P*-value
All-cause death	65 (53.3)	25 (86.2)	<*0.001*	15 (22.4)	65 (53.3)	<*0.001*	65 (53.3)	25 (86.2)	*0.001*
Endpoint events	94 (77.0)	26 (89.7)	<*0.001*	31 (46.3)	94 (77.0)	<*0.001*	94 (77.0)	26 (89.7)	*0.131*
*Non-fatal stroke*	4 (3.3)	3 (10.3)	*0.16*	2 (3.0)	4 (3.3)	*0.912*	4 (3.3)	3 (10.3)	*0.13*
*GIH*	17 (13.9)	4 (13.8)	*0.066*	2 (3.0)	17 (13.9)	*0.017*	17 (13.9)	4 (13.8)	*0.984*
*AVB*	24 (19.7)	4 (13.8)	*0.75*	8 (11.9)	24 (19.7)	*0.175*	24 (19.7)	4 (13.8)	*0.464*
*VT/VF*	23 (18.9)	9 (31.0)	*0.069*	10 (14.9)	23 (18.9)	*0.496*	23 (18.9)	9 (31.0)	*0.149*

GIH, gastrointestinal hemorrhage; AVB, atrioventricular block; VT/VF, ventricular tachycardia/ventricular fibrillation. The italicized terms are the *p*-values of comparison between the three cohorts.

In univariable analysis, a higher D-dimer level (per 1,000 ng/ml increase) was associated with an increased incidence of endpoint events (*HR* = 1.060, 95% CI 1.034–1.088, *p* < 0.001 and *HR* = 1.045, 95% CI 1.019–1.072, *p* < 0.001 for 30-day mortality and MACEs, respectively) (Table 3). After adjustments for confounding variates, D-dimer remained an independent risk factor in multivariable analysis. Compared with the low D-dimer level group, patients with medium and high levels of D-dimer showed a significantly higher risk of 30-day mortality (*HR* = 1.768, 95% CI 0.982–3.183, *p* = 0.057 and *HR* = 2.602, 95% CI 1.310–5.168, *p* = 0.006, respectively). A similar relationship was observed for MACEs. Compared with the low D-dimer level group, patients with medium and high levels of D-dimer also had a significantly higher risk of MACEs (*HR* = 2.012, 95% CI 1.329–3.044, *p* = 0.001 and *HR* = 2.543, 95% CI (1.452–4.453), *p* = 0.001, respectively) (Table 4).

**TABLE 3:** Univariable Cox regression analysis.

	30-day mortality			Secondary endpoint events		
	HR	95% CI	*P*-value	HR	95% CI	*P*-value
D-dimer (per 1,000 ng/ml increase)	1.06	(1.034–1.088)	<0.001	1.045	(1.019–1.072)	0.001
Low	1			1		
Median	2.996	(1.707–5.258)	<0.001	2.292	(1.525–3.444)	<0.001
High	6	(3.131–11.497)	<0.001	3.66	(2.155–6.216)	<0.001
Male sex	0.659	(0.446–0.973)	0.036	0.891	(0.638–1.244)	0.497
Age	1.044	(1.025–1.063)	<0.001	1.024	(1.009–1.039)	0.002
Body mass index	0.942	(0.882–1.007)	0.079	0.978	(0.923–1.036)	0.45
Alcohol use	0.632	(0.406–0.986)	0.043	0.808	(0.572–1.140)	0.224
Smoker	0.499	(0.337–0.741)	0.001	0.6	(0.435–0.827)	0.002
Hypertension	1.409	(0.955–2.080)	0.084	1.426	(1.034–1.966)	0.03
Diabetes mellitus	1.307	(0.88–1.941)	0.184	1.132	(0.810–1.582)	0.467
Coronary artery disease	1.187	(0.742–1.901)	0.475	1.327	(0.889–1.981)	0.166
Myocardial infarction	1.973	(0.995–3.913)	0.052	1.387	(0.707–2.721)	0.341
Percutaneous coronary intervention	1.837	(1.026–3.289)	0.041	2.009	(1.194–3.379)	0.009
Heart failure	1.244	(0.577–2.679)	0.578	1.133	(0.530–2.420)	0.748
Atrial fibrillation	1.06	(0.492–2.283)	0.882	1.15	(0.605–2.184)	0.67
Stroke	1.541	(0.802–2.958)	0.194	1.855	(1.068–3.222)	0.028
Valvular heart disease	0.919	(0.374–2.256)	0.853	0.889	(0.416–1.899)	0.762
Heart rate	1.005	(0.999–1.012)	0.122	1.005	(0.999–1.010)	0.123
Left ventricular ejection fraction < 50%	2.531	(1.67–3.834)	<0.001	1.655	(1.195–2.290)	0.002
White blood cell	1.114	(1.081–1.147)	<0.001	1.101	(1.072–1.132)	<0.001
Neutrophil	1.069	(1.047–1.092)	<0.001	1.047	(1.030–1.063)	<0.001
C reactive protein	1.018	(0.984–1.052)	0.301	1.017	(0.991–1.045)	0.198
Procalcitonin	1.004	(0.997–1.011)	0.269	1.002	(0.996–1.009)	0.486
Lactate	1.095	(1.063–1.128)	<0.001	1.073	(1.046–1.101)	<0.001
Troponin I	1.021	(1.002–1.041)	0.033	1.005	(0.988–1.022)	0.597
Albumin	0.956	(0.931–0.981)	0.001	0.974	(0.950–0.998)	0.033
Creatine	1.003	(1.002–1.005)	<0.001	1.005	(1.003–1.006)	<0.001
Bilirubin	1.019	(1.005–1.033)	0.006	1.019	(1.007–1.031)	0.002
Hemoglobin	0.993	(0.985–1.002)	0.127	0.996	(0.988–1.004)	0.314
White blood cell count	1.114	(1.081–1.147)	<0.001	1.101	(1.072–1.132)	<0.001
B-type natriuretic peptide > 400 pg/ml	3.637	(2.248–5.885)	<0.001	1.762	(1.258–2.467)	0.001
Fibrin	0.973	(0.857–1.104)	0.669	0.97	(0.873–1.078)	0.575
Prothrombin activity	0.982	(0.976–0.988)	<0.001	0.986	(0.980–0.991)	<0.001
International normalized ratio	1.127	(1.027–1.236)	0.012	1.135	(1.036–1.244)	0.007
Anticoagulants	0.774	(0.491–1.222)	0.272	0.994	(0.692–1.427)	0.974
Aspirin	0.742	(0.387–1.425)	0.37	0.733	(0.415–1.295)	0.285
P2Y12 inhibitor	0.371	(0.207–0.666)	0.001	0.426	(0.245–0.740)	0.002
Stent implantation	0.387	(0.262–0.570)	<0.001	0.524	(0.378–0.727)	<0.001
Intra-aortic balloon pump	0.83	(0.487–1.415)	0.494	0.813	(0.521–1.267)	0.36

**TABLE 4:** Multivariable Cox proportional hazard model.

	30-day mortality			Secondary endpoint events		
	HR	95% CI	*P*-value	HR	95% CI	*P*-value
Male sex			0.818			0.385
Age	1.023	(1.003–1.044)	0.024			0.323
Systolic blood pressure			0.147			0.236
Heart rate			0.534	1.006	(1.000–1.012)	0.046
Left ventricular ejection fraction < 50%			0.461			0.408
**D-dimer**						
Low	Control (*HR* = 1)			Control (*HR* = 1)		
Median	1.768	(0.982–3.183)	0.057	2.012	(1.329–3.044)	0.001
High	2.602	(1.310–5.168)	0.006	2.543	(1.452–4.453)	0.001
Lactate	1.086	(1.045–1.128)	<0.001	1.055	(1.022–1.089)	0.001
Troponin I	1.029	(1.010–1.048)	0.003			0.709
Creatine			0.067	1.004	(1.002–1.006)	<0.001
B-type natriuretic peptide > 400 pg/ml	2.656	(1.614–4.371)	<0.001			0.076
Anterior wall infarction			0.503			0.987
Stent implantation	0.593	(0.387–0.908)	0.016			0.122
P2Y12 inhibitor			0.193			0.054

**FIGURE 2:**
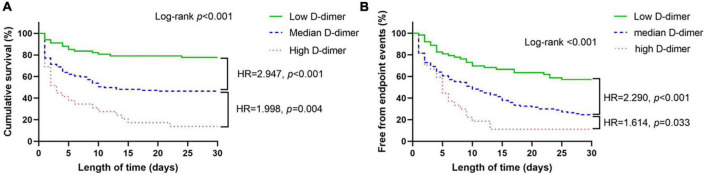
Continuous hazard ratio across D-dimer **(A)** and logarithmic D-dimer **(B)** according to restricted cubic spline analysis. *HR* = hazard ratio, line = predicted HR, dashed area = 95% confidence interval.

In Figure 2, we employed RCS to flexibly model and visualize the relationship of D-dimer level with all-cause mortality in the studied cohort. The RCS analysis demonstrated that the increase in D-dimer was constantly associated with higher all-cause mortality at relatively lower D-dimer levels (Figure 2A). However, the implications of extremely high D-dimer levels were discordant with those of low D-dimer levels. Nevertheless, after logarithmic transformation, the RCS curve inclined to be linear-like (Figure 2B).

### Improvement for risk discrimination by D-dimer

The discriminating abilities of external risk scores in our studied cohort were evaluated by C-index, the CardShock score (C-index 0.741, 95% CI 0.695–0.788), the IABP-SHOCK II score (C-index 0.732, 95% CI 0.679–0.786), and the GRACE score (C-index 0.679, 95% CI 0.628–0.730). After addition of D-dimer, the discriminating ability was improved with statistical significance (C-index: 0.756, 95% CI 0.711–0.801 *p* = 0.0.04, C-index: 0.754, 95% CI 0.705–0.803 *p* = 0.006, and C-index: 0.702, 95% CI 0.650–0.753 *p* < 0.001, respectively). Similar results were acquired when logarithmic D-dimer was included (C-index 0.756, 95% CI 0.710–0.802 *p* < 0.001, C-index 0.750, 95% CI 0.700–0.800 *p* < 0.001, and C-index 0.715, 95% CI 0.665–0.765 *p* < 0.001, respectively) (Table 5). The calibration curve showed good agreement between predicted and observed 30-day mortality in all of the abovementioned models (Supplementary Figures 1–4).

**TABLE 5:** Additional prognostic value of D-dimer for the primary outcome.

	CardShock score	IABP-SHOCK II score	GRACE score
**Original models**			
C-index	0.741 (0.695–0.788)	0.732 (0.679–0.786)	0.679 (0.628–0.730)
**Original models + D-dimer (high, median, low)**			
C-index	0.756 (0.711–0.801)	0.754 (0.705–0.803)	0.702 (0.650–0.753)
*p* difference	0.004	0.006	<0.001
**Original models + logarithmic D-dimer**			
C-index	0.756 (0.710–0.802)	0.750 (0.700–0.800)	0.715 (0.665–0.765)
*p* difference	<0.001	<0.001	<0.001

### The increment of risk reclassification by D-dimer

**FIGURE 3:**
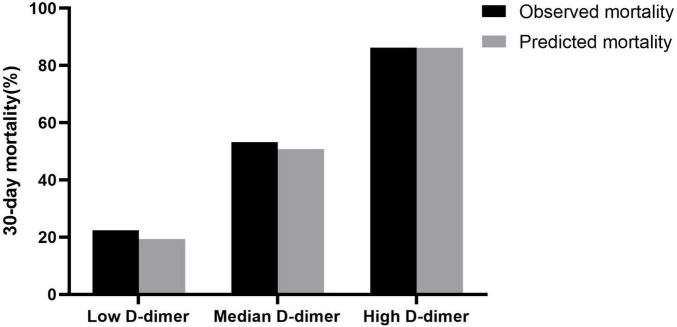
Predicted and observed mortality according to low (<720 ng/ml), median (720–3,600 ng/ml), and high (>3,600 ng/ml) levels of D-dimer at 30 days.

Using the risk prediction models combined with established risk factors and stratified D-dimer level, we calculated the predicted 30-day mortality and compared it with the actual observations (Figure 3). The average predicted mortality of the high D-dimer level was well-matched with the observed mortality. A similar result was observed for the median and low D-dimer cohorts, and the rates of concordance were 91.0, 77.0, and 86.2% for patients with high, medium, and low D-dimer, respectively. The net reclassification index was calculated for improvements in risk predictions after addition of plasma D-dimer, and the inclusion of D-dimer allowed more patients to be reclassified into more appropriate risk profiles (8.6% in IABP-SHOCK II score, 7.5% in CardShock score, and 12.8% in GRACE score. Detailed information is available in Supplementary Table 2.

## Discussion

The main findings from our present study are as follows. First, admission D-dimer level was independently associated with short-term outcomes in patients with CS complicating AMI. Second, adding D-dimer to the traditional risk models could improve the predictive power. Third, the predictive improvements in risk models were consistent with the data in the real world. To the best of our knowledge, this is the first study to evaluate the association of admission D-dimer with short-term outcomes in patients with CS complicating AMI.

Sepsis, trauma, and myocardial injury are three major causes contributing to shock. Regardless of the initial insult, shock always involves tissue dysoxia, slow capillary flow, and endothelial damage. Damage-associated molecular patterns (DAMPs) released by injured cells, such as histones, mitochondrial DNA, and cell-free DNA, combined with pattern recognition receptors (toll-like receptors, nucleotide-binding oligomerization domain-like receptors, etc.) trigger the signal transduction pathway, leading to expression of inflammatory cytokines. Among all those inflammatory factors, tumor necrosis factor-α (TNF-α) and Interleukin-6 (IL-6) are recognized as major contributors to thrombin generation (19). Inflammatory response with the release of inflammatory factors induced expression of tissue factor on monocytic cells and endothelial cells (20), leading to thrombin generation.

In patients with AMI, unstable plaque ruptures and exposes subendothelial components to blood flow. Highly prothrombotic proteins, including von Willebrand factor and tissue factor, trigger platelet activation and coagulation cascade, further inducing local thrombus formation and homologous myocardial ischemia. Severe cardiac ischemia and subsequent myocardial necrosis lead to profound depression of cardiac contractility and deterioration of cardiac compensation, resulting in a deleterious spiral of reduced cardiac output, low blood pressure, and further coronary ischemia, followed by an additional reduction in contractility (21). In the setting of AMI complicated by CS, systemic activation of inflammation and coagulation occur, resulting in microcirculatory deterioration. Moreover, inflammation and coagulation interact with each other, resulting in a vicious circle. In addition, inflammatory mediators such as Interleukin-6 (IL-1), IL-6, and TNF-α, are released, aggravating coagulation and endothelial damage. The fibrinolytic system is also activated, followed by plasmin digestion of fibrin which results in the generation of D-dimer. Therefore, an elevated D-dimer level indicates procoagulant activity and ongoing fibrinolysis.

The association of D-dimer with the outcome in patients with coronary artery disease has been widely established. The LIPID trial which enrolled patients with stable coronary heart disease demonstrated that higher D-dimer levels were associated with an increased risk of death after a median of 6 years of follow-up (10). Furthermore, the D-dimer level was found to be higher in patients with ST-elevation myocardial infarction (STEMI) than in those with chronic coronary disease or healthy individuals (22). A biomarker sub-study of HORIZONS-AMI also found that D-dimer ≥0.71 μg/ml measured at admission was associated with higher mortality within 3 years follow-up in patients with AMI (23), indicating an ongoing thrombotic and fibrinolytic process during atherogenesis. Therefore, D-dimer is a reliable marker to predict the outcome in patients with coronary heart disease.

Several mechanisms may explain the predictive value of D-dimer for endpoint events in patients with CS complicating AMI. Firstly, elevated admission D-dimer reflected the severity of activated inflammation, coagulation, and fibrinolysis. Secondly, the restoration of patency in coronary arteries by primary PCI may fail to achieve the restoration of tissue perfusion, known as the no-reflow phenomenon, which is an independent predictor of worse outcomes. Ayhan et al. (24) demonstrated that the D-dimer level on admission independently predicted the occurrence of no-reflow after PCI. Among multiple factors involved in no-reflow, a high thrombus burden was well-accepted as one of the strongest factors. Recently, a meta-analysis written by Biccirè et al. (25) demonstrated that in patients with acute coronary syndrome (ACS), D-dimer level was not only positively associated with higher in-hospital and short/long-term complications, but also positively correlated with the no-reflow phenomenon, indicating that D-dimer was a useful marker to identify patients with residual thrombotic risk after ACS. Our present study extended previous findings and demonstrated the prognostic value of D-dimer in patients with CS-complicating AMI. Moreover, previous studies have shown that D-dimer levels reflect clot degradability (26). A higher D-dimer level might indicate a relatively unstable thrombus structure and susceptibility to lysis (26, 27). During interventions on coronary arteries, thrombotic particles occur due to the fragmentation of materials in the culprit lesion (28, 29). Mobilization of thrombotic material and plaque debris could cause distal embolism. A previous study analyzed the components of thrombus aspirated from patients undergoing PCI for STEMI and found that in patients with distal embolization, the clots contained more erythrocyte components, along with a bigger size of clots (30, 31). Clots rich in erythrocytes, known as “red clots,” were characterized by unstable features and worse clinical outcomes (32, 33). Thus, it is feasible that patients with higher D-dimer levels are inclined to be those with “red clots.” Therefore, elevated D-dimer levels may indirectly reflect the thrombus size and components.

In our present study, the non-survivors were older and presented with lower systolic blood pressure and a higher level of lactate, B-type natriuretic peptide, white blood cell count, creatinine, risk scores (CardShock, IABP-SHOCK II, GRACE), but had lower left ventricular ejection fraction and stenting rate, which are all risk factors associated with poor outcome. Notably, nutritional indices are also important prognostic factors. Bicciré et al. (34) recently demonstrated that a low albumin level was associated with worse in-hospital adverse events including CS, resuscitated cardiac arrest, and death in patients with STEMI. Although the albumin level was associated with outcome in the univariable analysis, it was not found to be an independent risk factor in the present study following multivariable analysis. The inconsistency between our study and the study referred to may possibly be due to different inclusion criteria between the two studies. Our present study focused on the CS complicating AMI, while Bicciré et al. enrolled STEMI patients. However, the sample size in both studies was relatively small, and more studies are still warranted to clarify the prognostic value of albumin levels in patients with ACS complicated by CS.

Several models have been established to evaluate the outcome in patients with CS-complicating AMI, such as the IABP-SHOCK II score and CardShock score (17, 18); however, these models had only modest discrimination power, whereas the addition of D-dimer to these traditional models further improved the discrimination power in our study. Moreover, the predictive models were consistent with real practice, underscoring the improved utility of adding D-dimer to the traditional predictive models. Indeed, previous studies have shown that the D-dimer level could predict both the development of heart failure and the outcome in patients with AMI (7, 8). According to our present findings, D-dimer was not only a risk factor but also a predictor for the outcome in patients with CS complicating AMI.

Our present study has some strength in daily practice. First, as a simple testing, D-dimer could provide useful information for risk stratification in patients with CS-complicating AMI. Moreover, the addition of D-dimer to traditional risk models further improved the predictive power. Therefore, D-dimer should be regarded as a factor taking into current risk models. Second, previous studies have shown that the administration of anticoagulation therapy could reduce the D-dimer level (35). Whether reduction of D-dimer by anticoagulation therapy is associated with reduced risk in CS, will require further investigation.

### Limitation

As a single-center, small sample size, and retrospective observational study, our present study has some inherent limitations. First, the significant difference in revascularization rate between survivors and non-survivors could cause potential bias, as revascularization is strongly recommended for patients with AMI complicated with CS according to current guidelines (36). Due to the complex lesions or a serious critical condition some patients could not endure a revascularization procedure. The low revascularization rate also indicated a more severe status in non-survivors. Although we adjusted for this confounding factor, it may still cause potential bias. Second, the D-dimer level can be influenced by many clinical conditions such as active infections or pro-thrombotic states. Although the enrolled patients had no tumors or autoimmune diseases, other potential co-existing diseases may cause elevated D-dime. Moreover, whether an active infection and AMI coexisted cannot be completely excluded, because in the setting of AMI or AMI complicated by CS, inflammatory indicators, such as neutrophil and C-reactive protein, are usually elevated and may cause potential confounding effects. In addition, we only collected the D-dimer data at admission and did not perform a series of testing, which may provide more information for the association of D-dimer with the outcome. Therefore, more studies are still warranted to confirm our findings.

## Conclusion

Admission D-dimer was an independent risk factor associated with short-term outcome in patients with CS complicating AMI and the addition of D-dimer brought incremental risk prediction value to traditional risk prediction scores.

## Data availability statement

The datasets presented in this article are not readily available because based on the privacy policy of our institution, application for the dataset should not be granted at this time. Requests to access the datasets should be directed to BH, huangbi120@163.com.

## Ethics statement

The studies involving human participants were reviewed and approved by the Ethics Committee of the First Affiliated Hospital of Chongqing Medical University. The patients/participants provided their written informed consent to participate in this study. Written informed consent was obtained from the individual(s) for the publication of any potentially identifiable images or data included in this article.

## Author contributions

YJ completed all the analyses, writing, and figure/table development. SL and BH audited all the analyses, writing, and figure/table development. BS, GM, SC, YW, YZ, and ZX contributed advice and expertise on programming, data collection, and editorial support. All authors contributed to the article and approved the submitted version.
