# Supplementary material for: The prognostic value of admission D-dimer level in patients with cardiogenic shock after acute myocardial infarction
Source: Front Cardiovasc Med. 2023 Jan 9;9:1083881. doi: 10.3389/fcvm.2022.1083881 (PMC9868698; doi:10.3389/fcvm.2022.1083881)
Supplement: Supplementary file 1 [file Data_Sheet_1.docx]

**Supplementary figure 1.** Calibration curves for Cox regression models including D-dimer levels (high, median, and low) for 30-day all-cause mortality.


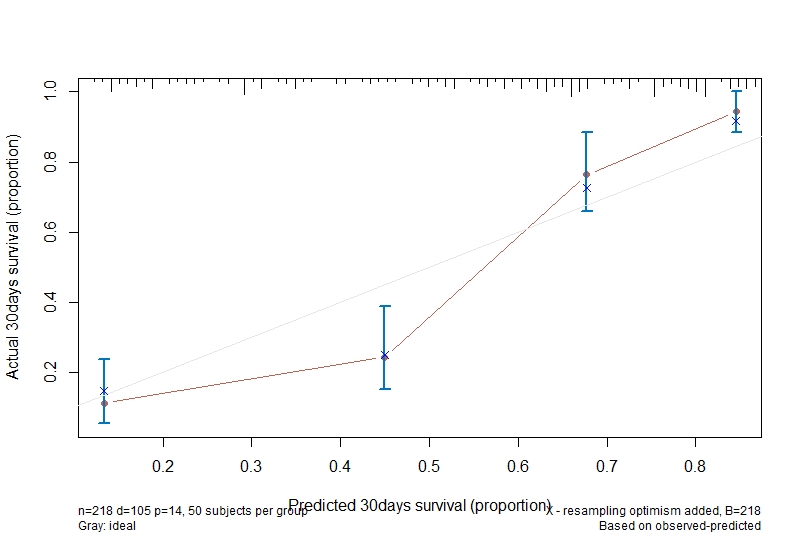


**Supplementary figure 2.** Calibration curves for Cardshock risk score including D-dimer levels (high, median, and low) for 30-day all-cause mortality.


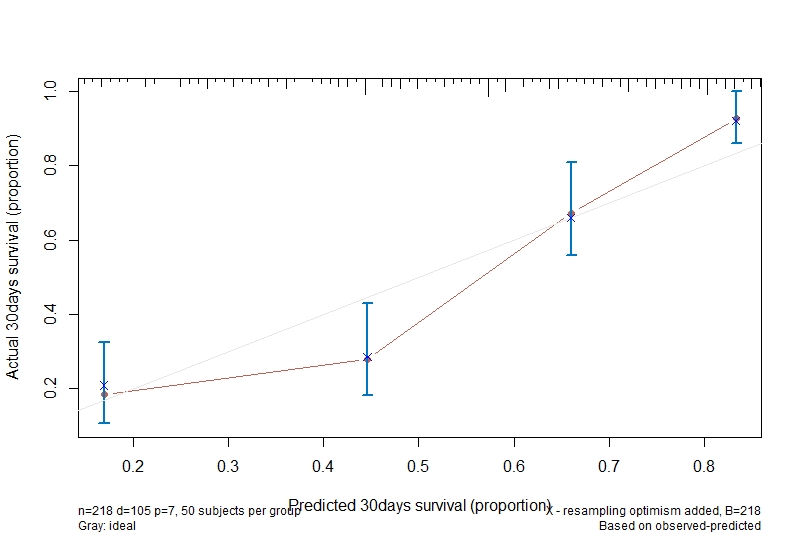


**Supplementary figure 3.** Calibration curves for IABP-SHOCK II risk score including D-dimer levels (high, median, and low) for 30-day all-cause mortality.


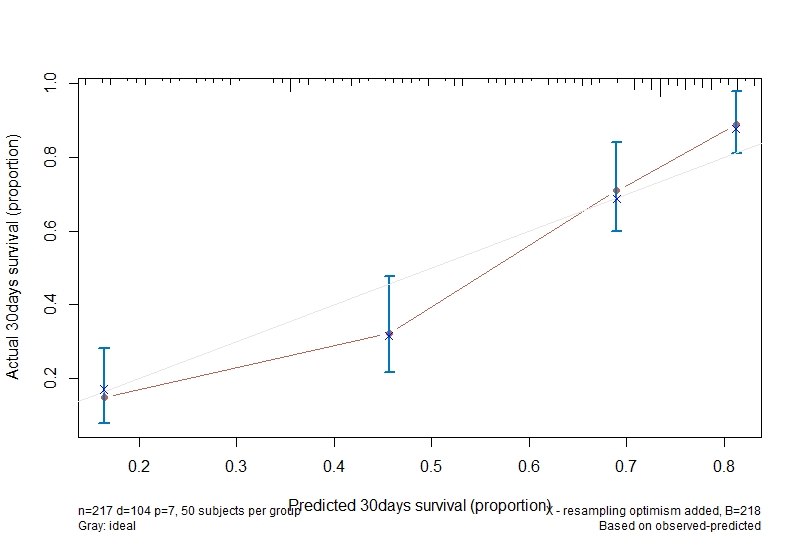


**Supplementary figure 4.** Calibration curves for GRACE risk score including D-dimer levels (high, median, and low) for 30-day all-cause mortality.


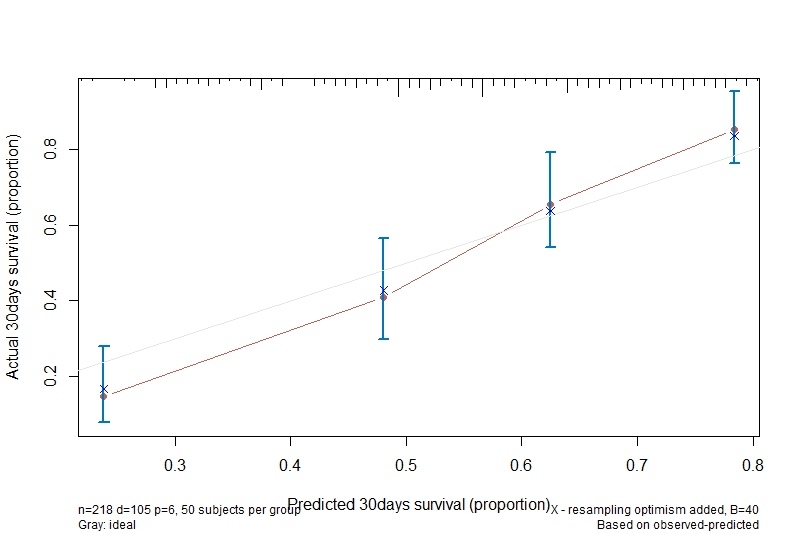


| **Table S1 Comparison of baseline characteristics stratified by D-dimer level** | | | | | | | |  |  |
| --- | --- | --- | --- | --- | --- | --- | --- | --- | --- |
|  | D-dimer<720 vs>3600ng/ml | | *p-value* | D-dimer <720 vs 720-3600 ng/ml | | *p-value* | D-dimer 720-3600 vs >3600 ng/ml | | *p-value* |
|  | low  D-dimer | high  D-dimer | | low  D-dimer | median  D-dimer |  | median  D-dimer | high  D-dimer |  |
| Age (Years) | 66.6±12.5 | 73.1±10.6 | *0.016* | 66.6±12.5 | 71.5±10.5 | *0.005* | 71.5±10.5 | 73.1±10.6 | *0.468* |
| Men | 45 (67.2%) | 14 (48.3%) | *0.081* | 45 (67.2%) | 82 (67.2%) | *0.995* | 82 (67.2%) | 14 (48.3%) | *0.057* |
| BMI (Kg/m2) | 23.3±2.9 | 23.0±3.8 | *0.668* | 23.3±2.9 | 23.0±2.8 | *0.493* | 23.0±2.8 | 23.0±3.8 | *0.958* |
| Alcohol use | 26 (38.8%) | 6 (20.7%) | *0.084* | 26 (38.8%) | 40 (32.8%) | *0.406* | 40 (32.8%) | 6 (20.7%) | *0.203* |
| Smoker | 41 (61.2%) | 10 (34.5%) | *0.016* | 41 (61.2%) | 64 (52.5%) | *0.248* | 64 (52.5%) | 10 (34.5%) | *0.082* |
| **Medical history** | | | | | | | | | |
| Hypertension | 30 (44.8%) | 13 (44.8) | *0.996* | 30 (44.8%) | 66 (62.0%) | *0.220* | 66 (54.1%) | 13 (44.8%) | *0.369* |
| Diabetes | 16 (23.9%) | 10 (34.5%) | *0.283* | 16 (23.9%) | 47 (38.5%) | *0.041* | 47 (38.5%) | 10 (34.5%) | *0.687* |
| CAD | 15 (22.4%) | 4 (13.8%) | *0.332* | 15 (22.4%) | 21 (17.2%) | *0.386* | 21 (17.2%) | 4 (13.8%) | *0.786* |
| MI | 3 (4.5%) | 2 (6.9%) | *0.636* | 3 (4.5%) | 7 (5.7%) | *0.711* | 7 (5.7%) | 2 (6.9%) | *0.683* |
| PCI | 6 (9.0%) | 1 (3.4%) | *0.671* | 6 (9.0%) | 11 (9.0%) | *0.989* | 11 (9.0%) | 1 (3.4%) | *0.463* |
| CABG | 0 | 0 | *-* | 0 | 3 (2.9%) | *0.553* | 3 (2.5%) | 0 | *0.394* |
| Stroke | 3 (4.5%) | 3 (10.3%) | *0.362* | 3 (4.5%) | 9 (7.4%) | *0.544* | 9 (7.4%) | 3 (10.3%) | *0.701* |
| Atrial fibrillation | 1 (1.5%) | 3 (10.3%) | *0.081* | 1 (1.5%) | 9 (7.4%) | *0.101* | 9 (7.4%) | 3 (10.3%) | *0.701* |
| Heart failure | 2 (3.0%) | 3 (10.3%) | *0.160* | 2 (3.0%) | 6 (4.9%) | *0.714* | 6 (4.9%) | 3 (10.3%) | *0.375* |
| **Vital signs** |  |  |  |  |  |  |  |  |  |
| SBP (mmHg) | 87.0 (80.0-91.0) | 85.0 (78.0-89.0) | *0.162* | 87.0 (80.0-91.0) | 85.0 (78.0-96.5) | *0.602* | 85.0 (78.0-96.5) | 85.0 (78.0-89.0) | *0.328* |
| DBP (mmHg) | 55.0 (51.0-61.0) | 55.0 (48.5-63.0) | *0.767* | 55.0 (51.0-61.0) | 56.5 (49.8-63.0) | *0.724* | 56.5 (49.8-63.0) | 55.0 (48.5-63.0) | *0.575* |
| Heart rate (bpm) | 90.0 (68.0-100.0) | 86.0 (59.5-114.5) | *0.848* | 90.0 (68.0-100.0) | 86.0 (68.8-110.0) | *0.905* | 86.0 (68.8-110.0) | 86.0 (59.5-114.5) | *0.755* |
| **Myocardial infarction location** | | | | | | | | | |
| Anterior | 27 (40.3%) | 10 (34.5%) | *0.591* | 27 (40.3%) | 43 (35.2%) | *0.491* | 43 (35.2%) | 10 (34.5%) | *0.938* |
| Inferior or posterior | 29 (43.3%) | 12 (41.4%) | *0.863* | 29 (43.3%) | 53 (43.4%) | *0.983* | 53 (43.4%) | 12 (41.4%) | *0.840* |
| Lateral | 7 (10.4%) | 3 (10.3%) | *0.988* | 7 (10.4%) | 8 (6.6%) | *0.344* | 8 (6.6%) | 3 (10.3%) | *0.443* |
| Right ventricle | 9 (13.4%) | 4 (13.8%) | *0.962* | 9 (13.4%) | 14 (11.5%) | *0.694* | 14 (11.5%) | 4 (13.8%) | *0.752* |
| **Laboratory and echocardiography finding** | | | | | | | | | |
| WBC (*109) | 8.4 (6.6-11.0) | 13.8 (9.3-21.3) | *<0.001* | 8.4 (6.6-11.0) | 11.6 (7.6-15.6) | *0.001* | 11.6 (7.6-15.6) | 13.8 (9.3-21.3) | *0.023* |
| Neutrophil (%) | 73.9±10.4 | 79.9±17.4 | *0.042* | 73.9±10.4 | 79.4±10.9 | *0.001* | 79.4±10.9 | 79.9±17.4 | *0.854* |
| Procalcitonin  (μg/L) | 0.06 (0.05-0.45) | 0.84 (0.13-6.24) | *0.180* | 0.06 (0.05-0.45) | 0.53 (0.05-2.54) | *0.080* | 0.53 (0.05-2.54) | 0.84 (0.13-6.24) | *0.261* |
| CRP  (mg/L) | 13.8±7.0 | 16.1±7.1 | *0.129* | 13.8±7.0 | 14.7±7.1 | *0.217* | 14.7±7.1 | 16.1±7.1 | *0.474* |
| Lactate (mmol/L) | 2.4 (1.8-3.8) | 6.0 (2.7-8.9) | *<0.001* | 2.4 (1.8-3.8) | 4.1 (2.1-7.0) | *0.001* | 4.1 (2.1-7.0) | 6.0 (2.7-8.9) | *0.034* |
| TNI  (ng/mL) | 4.9 (1.0-11.2) | 5.3 (1.5-12.2) | *0.722* | 4.9 (1.0-11.2) | 4.1 (0.7-9.6) | *0.598* | 4.1 (0.7-9.6) | 5.3 (1.5-12.2) | *0.483* |
| BNP (pg/mL) | 346.0 (78.6-1563.0) | 2352 (620.5-4176.0) | *<0.001* | 346.0 (78.6-1563.0) | 888.5 (170.8-2826.8) | *0.014* | 888.5 (170.8-2826.8) | 2352 (620.5-4176.0) | *0.015* |
| D-dimer (ng/mL) | 300 (155-550) | 680 (467-1407) | *<0.001* | 300 (155-550) | 1520 (945-2330) | *<0.001* | 1520 (945-2330) | 680 (467-1407) | *<0.001* |
| Albumin (g/L) | 35.8±6.2 | 31.1±5.9 | *0.001* | 35.8±6.2 | 33.7±6.5 | *0.026* | 33.7±6.5 | 31.1±5.9 | *0.053* |
| Creatinine (umol/L) | 90.0 (68.0-124.0) | 154.0 (96.0-222.5) | *<0.001* | 90.0 (68.0-124.0) | 116.5 (88.8-179.5) | *0.001* | 116.5 (88.8-179.5) | 154.0 (96.0-222.5) | *0.131* |
| LVEF(%) | 52±8.6 | 44.0±9.2 | *<0.001* | 52±8.6 | 47.4±10.0 | *0.001* | 47.4±10.0 | 44.0±9.2 | *0.089* |
| PTA (%) | 99.0 (84.0-114.8) | 64.6 (50.0-81.7) | *<0.001* | 99.0 (84.0-114.8) | 83.1 (63.9-98.6) | *<0.001* | 83.1 (63.9-98.6) | 64.6 (50.0-81.7) | *0.005* |
| **Risk scores** |  |  |  |  |  |  |  |  |  |
| CardShock | 4 (3-5) | 4 (4-6) | *0.010* | 4 (3-5) | 4 (3-5) | *0.010* | 4 (3-5) | 4 (4-6) | *0.452* |
| GRACE | 211.5±25.5 | 232.0±25.8 | *<0.001* | 211.5±25.5 | 224.0±22.9 | *0.001* | 224.0±22.9 | 232.0±25.8 | *0.101* |
| IABP-SHOCK II | 2 (1-3) | 4 (3-6) | *<0.001* | 2 (1-3) | 3 (2-4) | *0.001* | 3 (2-4) | 4 (3-6) | *0.055* |
| **Treatment** |  |  |  |  |  |  |  |  |  |
| Aspirin | 64 (95.9%) | 24 (82.8%) | *0.052* | 64 (95.5%) | 113 (92.6%) | *0.544* | 113 (92.6%) | 24 (82.8%) | *0.146* |
| P2Y12 inhibitor | 66 (98.5%) | 26 (89.7%) | *0.081* | 66 (98.5%) | 111 (91.0%) | *0.059* | 111 (91.0%) | 26 (89.7%) | *0.733* |
| Dopamine | 27 (40.3%) | 18 (62.1%) | *0.05* | 27 (40.3%) | (71 58.2%) | *0.018* | 71 (58.2%) | 18 (62.1%) | *0.703* |
| Nitrates | 22 (32.8%) | 5 (17.2%) | *0.119* | 22 (32.8%) | 36 (29.5%) | *0.635* | 36 (29.5%) | 5 (17.2%) | *0.182* |
| Digitails | 6 (9.0%) | 3 (10.3%) | *0.830* | 6 (9.0%) | 19 (15.6%) | *0.199* | 19 (15.6%) | 3 (10.3%) | *0.572* |
| Stenting | 57 (85.1%) | 15 (51.7%) | *0.001* | 57 (85.1%) | 73 (59.8%) | *<0.001* | 73 (59.8%) | 15 (51.7%) | *0.426* |
| IABP | 8 (11.9%) | 4 (13.8%) | *0.750* | 8 (11.9%) | 24 (19.7%) | *0.175* | 24 (19.7%) | 4 (13.8%) | *0.464* |
| Ventilation support | 18 (26.9%) | 18 (62.1%) | *0.001* | 18 (26.9%) | 62 (50.8%) | *0.001* | 62 (50.8%) | 18 (62.1%) | *0.275* |
| Hemofiltration | 0 | 1 (3.4%) | *0.302* | 0 | 2 (1.6%) | *0.540* | 2 (1.6%) | 1 (3.4%) | *0.475* |
| Anticoagulation | 22 (32.8%) | 6 (20.7%) | *0.229* | 22 (32.8%) | 29 (23.8%) | *0.179* | 29 (23.8%) | 6 (20.7%) | *0.724* |

**Table S2 Net reclassification index (NRI) with addition of information on D-dimer**

|  | IABP-SHOCK II score | CardShock score | GRACE score |
| --- | --- | --- | --- |
| Case/died (N=105) | 7.70% | 6.60% | 4.80% |
| non-case/survival (N=113) | 0.90% | 0.90% | 8.00% |
| Additional NRI | 8.60% | 7.50% | 12.8% |
